# Dissecting Bacterial Cell Wall Entry and Signaling in Eukaryotic Cells: an Actin-Dependent Pathway Parallels Platelet-Activating Factor Receptor-Mediated Endocytosis

**DOI:** 10.1128/mBio.02030-16

**Published:** 2017-01-03

**Authors:** Lip Nam Loh, Geli Gao, Elaine I. Tuomanen

**Affiliations:** Department of Infectious Diseases, St. Jude Children’s Research Hospital, Memphis, Tennessee, USA; Emory University; University of Mississippi Medical Center

## Abstract

The Gram-positive bacterial cell wall (CW) peptidoglycan-teichoic acid complex is released into the host environment during bacterial metabolism or death. It is a highly inflammatory Toll-like receptor 2 (TLR2) ligand, and previous *in vivo* studies have demonstrated its ability to recapitulate pathological features of pneumonia and meningitis. We report that an actin-dependent pathway is involved in the internalization of the CW by epithelial and endothelial cells, in addition to the previously described platelet-activating factor receptor (PAFr)-dependent uptake pathway. Unlike the PAFr-dependent pathway, which is mediated by clathrin and dynamin and does not lead to signaling, the alternative pathway is sensitive to 5-(*N*-ethyl-*N*-isopropyl) amiloride (EIPA) and engenders Rac1, Cdc42, and phosphatidylinositol 3-kinase (PI3K) signaling. Upon internalization by this macropinocytosis-like pathway, CW is trafficked to lysosomes. Intracellular CW trafficking is more complex than previously recognized and suggests multiple points of interaction with and without innate immune signaling.

## IMPORTANCE

*Streptococcus pneumoniae* is a major human pathogen infecting the respiratory tract and brain. It is an established model organism for understanding how infection injures the host. During infection or bacterial growth, bacteria shed their cell wall (CW) into the host environment and trigger inflammation. A previous study has shown that CW enters and crosses cell barriers by interacting with a receptor on the surfaces of host cells, termed platelet-activating factor receptor (PAFr). In the present study, by using cells that are depleted of PAFr, we identified a second pathway with features of macropinocytosis, which is a receptor-independent fluid uptake mechanism by cells. Each pathway contributes approximately the same amount of cell wall trafficking, but the PAFr pathway is silent, while the new pathway appears to contribute to the host inflammatory response to CW insult.

## INTRODUCTION

The Gram-positive bacterial cell wall (CW) is composed of a network of peptidoglycan decorated by teichoic acids and lipoteichoic acids. During infection, CW components traffic throughout the body, either as intact bacteria or as components released during bacterial metabolism or death, and cross endothelial and epithelial barriers (1, 2). They are also released by the microbiome and circulate in the bloodstream (3). CW components are highly inflammatory in many models of infection, a property conveyed by recognition by the innate immune receptor Toll-like receptor 2 (TLR2) on the plasma membranes of phagocytes and epithelial and endothelial cells (4–7). TLR2 signaling results in activation of p38 mitogen-activated protein kinase, NF-κB, and the secretion of proinflammatory cytokines, such as tumor necrosis factor alpha (TNF-α), interleukin 1 (IL-1), and IL-6 (7–10). Ingestion of CW by professional phagocytes leads to digestion by lysozyme, resulting in products in the cytoplasm that can be sensed by the nucleotide-binding oligomerization domain 2 (Nod2), which leads to the release of the chemokine CCL2 (CC motif chemokine ligand 2) for recruiting macrophages to the site of infection (11).

While CW signaling paradigms are well established, it is much less well understood how CW enters nonphagocytic cells and crosses epithelial and endothelial barriers as it traffics through the host. One known route of CW uptake into eukaryotic cells is shared by respiratory pathogens that present phosphorylcholine (PCho) on their surfaces (1, 12, 13). PCho is the bioactive component of the chemokine platelet-activating factor (PAF), and by molecular mimicry, bacteria bearing PCho bind to the G-protein-coupled PAF receptor (PAFr) and trigger β-arrestin-mediated internalization into host cells (12–14). In the case of pneumococcus, a major respiratory pathogen, PCho is presented as a covalent adduct to CW, and similar to intact living bacteria, purified pneumococcal CW enters cells and crosses cellular barriers in a PAFr-dependent manner (1). However, once inside cells, the downstream endocytic pathway in the cytoplasm is unclear. PAFr-associated endocytosis, like other G-protein-coupled receptors (GPCR), has been reported to be clathrin dependent (15, 16). Recently, Boucrot et al. reported that several GPCR ligands are endocytosed via a newly described clathrin-independent pathway, termed fast endophilin-mediated endocytosis (FEME), which has no known role in pathogen endocytosis yet (17). The relevance of these pathways to CW uptake is unknown. Thus, the details of how PCho ligands from a major group of pathogens traffic in host cells is unclear.

Most nonrespiratory pathogens do not display PCho on the CW, and thus, non-PAFr endocytic pathways for CW uptake must exist. These other pathways have been suggested but not studied in detail (18). Therefore, we embarked on a detailed analysis of not only the PAFr endocytic pathway but also other possible CW uptake pathways. We report that TLR2 is responsible for most CW inflammatory signaling, but not CW uptake. CW internalization via PAFr is clathrin and dynamin dependent, contributes to ~40% of endocytic events, and results in virtually no downstream inflammatory signaling. Independent of and in parallel with PAFr, CW internalization is also mediated by an actin-dependent pathway, which results in Rac1, Cdc42, and phosphatidylinositol 3-kinase (PI3K) signaling. This additional pathway appears to generate host cell signaling in conjunction with lysosomes.

## RESULTS

### TLR2, but not PAFr, initiates a CW-induced inflammatory response.

**FIG 1:**
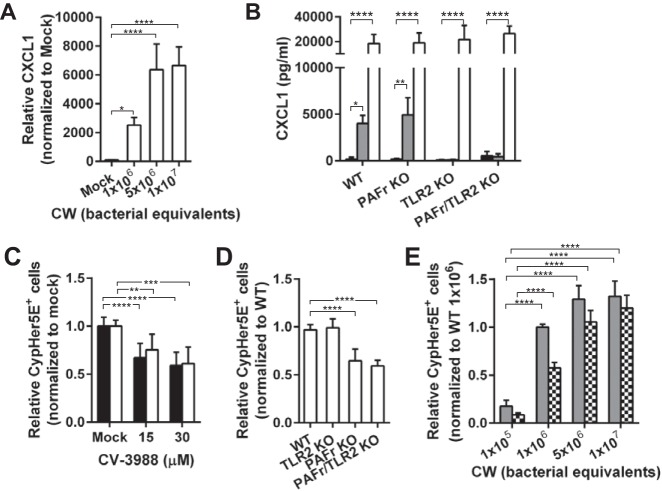
Distinct roles of PAFr and TLR2 during CW exposure. (A) Primary MLEC isolated from wild-type mice (WT MLEC) were incubated with doses of CW as indicated for 8 h at 37°C. The concentration of CXCL1 in the medium was analyzed by ELISA. Data are means plus standard deviations (SD) (error bars) from four independent experiments performed in triplicate. Values that are significantly different by Bonferroni’s multiple-comparison test are indicated by bars and asterisks as follows: *, *P* < 0.05; ****, *P* < 0.0001. (B) Primary MLEC from wild-type (WT), TLR2 knockout (TLR2 KO), PAFr knockout (PAFr KO), or PAFr/TLR2 double knockout (PAFr/TLR2 KO) mice were incubated with 10^7^ bacterial equivalents of CW (gray bars) or 5 µg/ml lipopolysaccharide (LPS) from *Escherichia coli* O111:B4 (white bars) for 8 h at 37°C. LPS, which is a TLR4 ligand, was used as a control to demonstrate that the cells were responsive to inflammation trigger. Untreated controls are depicted as black bars. CXCL1 production was analyzed by ELISA. Data are means plus SD from at least three independent experiments performed in triplicate. *, *P* < 0.05; **, *P* < 0.01; ****, *P* < 0.0001 (Bonferroni’s multiple-comparison test). (C) A549 cells (black bars) and HBMEC (white bars) were continuously treated with dimethyl sulfoxide (DMSO) (Mock) or CV-3988 at the concentration indicated. CypHer5E-labeled CW was added to cells for 2 h at 37°C, and the cells were washed and lifted from the well, and CypHer5E-positive cells were quantitated by flow cytometry. Data are presented as means plus SD from at least three independent experiments. **, *P* < 0.01; ***, *P* < 0.001; ****, *P* < 0.0001 (Bonferroni’s multiple-comparison test). (D) Primary MLEC isolated from WT or KO mice were incubated with CypHer5E-labeled CW, and CypHer5E-positive cells were analyzed as described above for panel C. Data are presented as means plus SD from at least three independent experiments. ****, *P* < 0.0001 (Bonferroni’s multiple-comparison test). (E) Primary MLEC from WT mice (solid gray bars) or PAFr KO mice (bars with checkerboard pattern) were incubated with the indicated doses of CypHer5E-labeled CW, and CypHer5E-positive cells were analyzed as described above for panel C. Data are presented as means plus SD from three independent experiments. ****, *P* < 0.0001 (Bonferroni’s multiple-comparison test).

PAFr and TLR2 are known to interact with intact pneumococci and purified pneumococcal peptidoglycan-teichoic acid complex (cell wall [CW]) on human brain microvascular endothelial cells (HBMEC) and human adenocarcinoma lung epithelial cells (A549) (7, 12). To study the functional roles of these receptors during CW exposure, we first treated mouse lung endothelial cells (MLEC) isolated from wild-type (WT) mice, with either 10^6^ or 10^7^ bacterial equivalents of purified CW for 8 h. We measured the secreted CXC chemokine ligand 1 (CXCL1), which was the most abundantly secreted CW-induced cytokine (see Fig. S1 in the supplemental material), in the medium by enzyme-linked immunosorbent assay (ELISA) (Fig. 1A). At the dosage of 1 × 10^6^ bacterial equivalents, CW induced significant secretion of CXCL1 that increased to a maximum plateau at ≥5 × 10^6^ bacterial equivalents. When MLEC isolated from PAFr knockout (PAFr KO) mice were incubated with 10^7^ bacterial equivalents of CW, CXCL1 secretion remained as high as in CW-treated WT cells. In contrast, the secreted CXCL1 in both TLR2 knockout (TLR2 KO), and PAFr/TLR2 double knockout (PAFr/TLR2 KO) MLEC remained at low baseline (Fig. 1B and Fig. S1). Extending the incubation of cells with CW for 24 h resulted in the upregulation of several other cytokines (including lipopolysaccharide-inducible CXC chemokine [LIX], IL-6, granulocyte-macrophage colony-stimulating factor [GM-CSF], G-CSF, macrophage inflammatory protein 1α [MIP-1α], and MIP-1γ) in addition to CXCL1 in WT, but not TLR2 KO, cells (Fig. S1). These data suggest that CW-induced inflammatory response is dependent on TLR2, but not PAFr.

10.1128/mBio.02030-16.1 Figure S1  Effects of CW treatment on cytokine expression. Representative array readouts of mouse cytokine production from WT (top) and TLR2 KO (bottom) MLEC treated with 10^7^ bacterial equivalents of CW or left untreated (Control) for the indicated time. The panel on the right shows the layout of the Mouse Inflammation Array C1 (Raybiotech). Download Figure S1, EPS file, 3.8 MB. Copyright © 2017 Loh et al. 2017 Loh et al. This content is distributed under the terms of the Creative Commons Attribution 4.0 International license.

### PAFr, but not TLR2, supports endocytosis of the CW into epithelial and endothelial cells.

To demonstrate the fate of CW upon ligation by PAFr, A549 cells and HBMEC were treated with and without PAFr antagonist CV-3988 and incubated with CW labeled with CypHer5E *N*-hydroxysuccinimide (NHS) ester (CypHer5E-labeled CW). As a measure of uptake into cells, induction of CypHer5E fluorescence by a change in pH upon entering endosomes was quantified by flow cytometry. Uptake was significantly reduced by ~50% in both A549 cells and HBMEC treated with CV-3988 compared to that in mock-treated cells (Fig. 1C).

To further test the involvement of TLR2 and PAFr in CW internalization, WT, PAFr KO, TLR2 KO, and PAFr/TLR2 KO MLEC were treated with CypHer5E-labeled CW. CW internalization in TLR2 KO MLEC was equivalent to that in WT MLEC, while internalization was reduced by ~40% in PAFr KO and PAFr/TLR2 KO cells (Fig. 1D). CW uptake by PAFr KO cells was dose dependent, similar to that in WT cells (Fig. 1E). These results confirmed that PAFr, but not TLR2, supported CW uptake.

The remaining ~50% uptake into PAFr KO cells indicated that a second CW endocytic pathway functioned in parallel to and independent of PAFr. To confirm that CW was internalized by cells independent of PAFr, WT and PAFr KO MLEC were incubated with fluorescein isothiocyanate (FITC)-labeled CW or ethanolamine-adapted CW (EA-CW), which does not contain PCho and is unable to bind PAFr. Extracellular CW was stained with anti-FITC sera without permeabilization. In agreement with the observation of CW uptake by PAFr KO cells in Fig. 1D, intracellular CW was confirmed in these cells (see Fig. S2B in the supplemental material). Also, intracellular EA-CW was observed in both WT and PAFr KO cells (Fig. S2C and S2D). The nature of this second receptor-independent pathway was analyzed further.

10.1128/mBio.02030-16.2 Figure S2  CW are internalized by MLEC PAFr KO. (A to D) WT MLEC (A and C) or PAFr KO MLEC (B and D) were incubated with FITC-labeled CW (A and B) or EA-CW (C and D) (green) as indicated for 2 h at 37°C, fixed with paraformaldehyde (PFA), and extracellular CW or EA-CW (magenta) were immunostained with goat anti-FITC sera without detergent permeabilization, followed by Alexa Fluor 647-conjugated chicken anti-goat sera. Cell nuclei were stained with DAPI (blue). Intracellular CW and EA-CW particles (arrows) were single color, and extracellular CW and EA-CW particles were double colors (green and magenta). A single confocal slice is shown. Bar, 10 µm. Download Figure S2, EPS file, 11.1 MB. Copyright © 2017 Loh et al. 2017 Loh et al. This content is distributed under the terms of the Creative Commons Attribution 4.0 International license.

### CW uptake involves two pathways that are distinguished by dynamin and clathrin.

**FIG 2:**
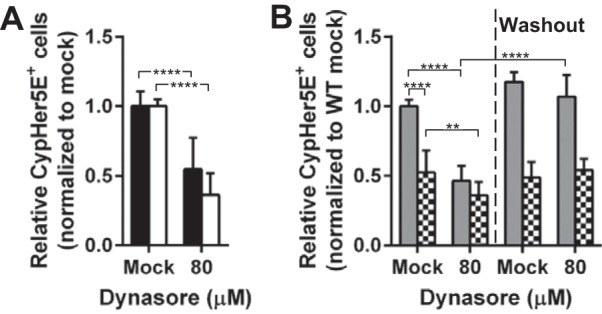
Role of dynamin in CW internalization. (A) A549 cells (black bars) and HBMEC (white bars) were pretreated with DMSO (Mock) or 80 µM dynasore, incubated with CypHer5E-labeled CW for 2 h at 37°C, and processed and analyzed as described in the legend to Fig. 1C. Data are presented as means plus SD from at least three independent experiments. ****, *P* < 0.0001 (Student’s *t* test). (B) WT MLEC (gray bars) and PAFr KO MLEC (checkerboard bars) were treated with CW and dynasore as described above for panel A (left) or dynasore was removed for the inhibitor washout experiment (right). Data are presented as means plus SD from three independent experiments. **, *P* < 0.01; ****, *P* < 0.0001 (Student’s *t* test).

Dynamin is a large GTPase involved in the scission and budding of vesicles from the plasma membrane in several endocytic pathways. To examine the requirement of dynamin in CW uptake, A549 cells and HBMEC were treated with dynasore (a dynamin inhibitor), followed by incubation with CypHer5E-labeled CW, and CW uptake was analyzed by flow cytometry. Uptake of transferrin (a positive marker for clathrin-mediated endocytosis which is dynamin dependent) by A549 and HBMEC treated with dynasore was reduced by 20 and 40%, respectively (see Fig. S5 in the supplemental material). Dynasore treatment resulted in 50% reduction of CW internalization by A549 cells, HBMEC, and primary WT MLEC compared to that in mock-treated cells (Fig. 2A and B). Although dynasore treatment led to a statistically significant reduction in the viability of WT MLEC (Fig. S7), the cells were still responsive to CW after the inhibitor was removed and CW uptake was similar to that in mock-treated cells (Fig. 2B). In PAFr KO MLEC, CW uptake remained largely intact in the presence of dynasore in comparison to mock-treated cells (Fig. 2B), suggesting that the novel PAFr-independent pathway was dynamin independent.

**FIG 3:**
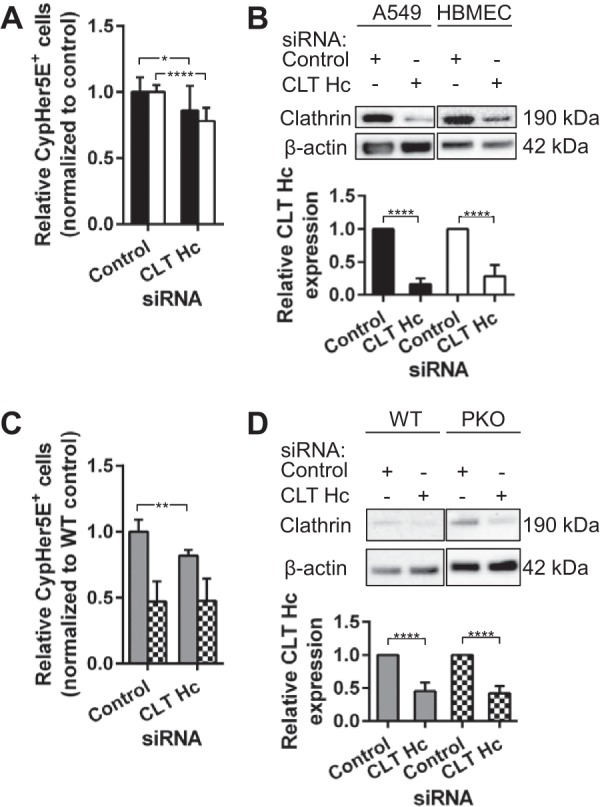
CW uptake via PAFr is clathrin dependent. (A) A549 cells (black bars) and HBMEC (white bars) transfected with siRNAs targeting clathrin heavy chain (CLT Hc) were incubated with CypHer5E-labeled CW and processed and analyzed as described in the legend to Fig. 1C. Data are presented as means plus SD from at least three independent experiments. *, *P* < 0.05; ****, *P* < 0.0001 (Student’s *t* test). (B, top) Representative Western blots showing the CLT Hc protein levels in cell lysates harvested from A549 cells and HBMEC transfected with siRNAs as described above for panel A. (Bottom) Densitometric quantification of CLT Hc expression level in cell lysates in top panel. Protein level was normalized to β-actin. ****, *P* < 0.0001 (Student’s *t* test). (C) WT MLEC (gray bars) and PAFr KO MLEC (checkerboard bars) transfected with siRNAs targeting CLT Hc were incubated with CypHer5E-labeled CW and processed and analyzed as described in the legend to Fig. 1C. Data are presented as means plus SD from at least three independent experiments. **, *P* < 0.01 (Student’s *t* test). (D, top) Representative Western blots showing the CLT Hc protein levels in cell lysates harvested from MLEC transfected with siRNAs as described above in the legend to panel C. (Bottom) Densitometric quantification of CLT Hc expression level in cell lysates in the top panel. Protein level was normalized to β-actin. ****, *P* < 0.0001 (Student’s *t* test). PKO, PAFr KO.

PAF induces clathrin-mediated endocytosis in neutrophils (15). To determine whether CW internalization by A549 cells and HBMEC was clathrin dependent, endogenous clathrin heavy chain was depleted with small interfering RNA (siRNA) (Fig. 3A and B). A549 and HBMEC endocytosis of CW was reduced by only 14 and 22%, respectively, while endogenous clathrin heavy chain was significantly depleted. In MLEC, clathrin and endophilin A2 depletion led to cell viability issues; hence, the siRNA transfection time was reduced to 36 h and resulted in approximately 50% knockdown efficiency (Fig. 3D; see Fig. S3A in the supplemental material). Despite the partial knockdown efficiency, CW uptake by WT cells was reduced by 18% and 19% for clathrin heavy chain and endophilin A2 knockdown, respectively; the knockdown had no effect on CW internalization by PAFr KO cells (Fig. 3C and Fig. S3A). Thus, we concluded that clathrin and endophilin appeared to play detectable but minor roles in CW uptake by the PAFr pathway. The PAFr pathway would appear to be distinct from the new clathrin-independent pathway termed fast endophilin-mediated endocytosis (FEME) reported by Boucrot et al. (2015) to mediate the internalization of various GPCR ligands (17).

10.1128/mBio.02030-16.3 Figure S3  CW internalization is independent of endophilin A2 and caveolin 1. (A, left) WT MLEC (gray bars) and PAFr KO MLEC (checkerboard bars) transfected with siRNAs targeting endophilin A2 were incubated with CypHer5E-labeled CW and processed and analyzed as described in the legend to Fig. 1C. Data are presented as mean ± SD from at least two independent experiments. **, *P* < 0.01 (Student’s *t* test). (Right) Quantification of endophilin A2 expression level in cell lysates harvested from cells transfected with siRNAs targeting endophilin A2 in the left panel. Protein level was normalized to β-actin. *, *P* < 0.05; ***, *P* < 0.001 (Student’s *t* test). (B and C, left) A549 cells (black bars), HBMEC (white bars), WT MLEC (gray bars), and PAFr KO MLEC (checkerboard bars) transfected with siRNAs targeting caveolin 1 (Cav1) were incubated with CypHer5E-labeled CW and processed and analyzed as described in the legend to Fig. 1C. Data are presented as means ± SD from at least two independent experiments. *, *P* < 0.05, ****, *P* < 0.0001 (Student’s *t* test). (Right) Quantification of Cav1 expression level in cell lysates harvested from cells transfected with siRNAs targeting Cav1 in the left panel. Protein level was normalized to β-actin. *, *P* < 0.05; **, *P* < 0.01; ***, *P* < 0.001 (Student’s *t* test). Download Figure S3, EPS file, 2.5 MB. Copyright © 2017 Loh et al. 2017 Loh et al. This content is distributed under the terms of the Creative Commons Attribution 4.0 International license.

Caveolae-mediated endocytosis, another dynamin-dependent pathway (19, 20), was investigated by depleting endogenous caveolin 1 (Cav1) by siRNA. No inhibition of CW internalization was observed (see Fig. S3B and S3C in the supplemental material). Thus, we concluded that CW uptake by PAFr was dependent on clathrin and dynamin, while the alternative endocytic pathway was largely independent of dynamin, clathrin, and caveolin.

**FIG 4:**
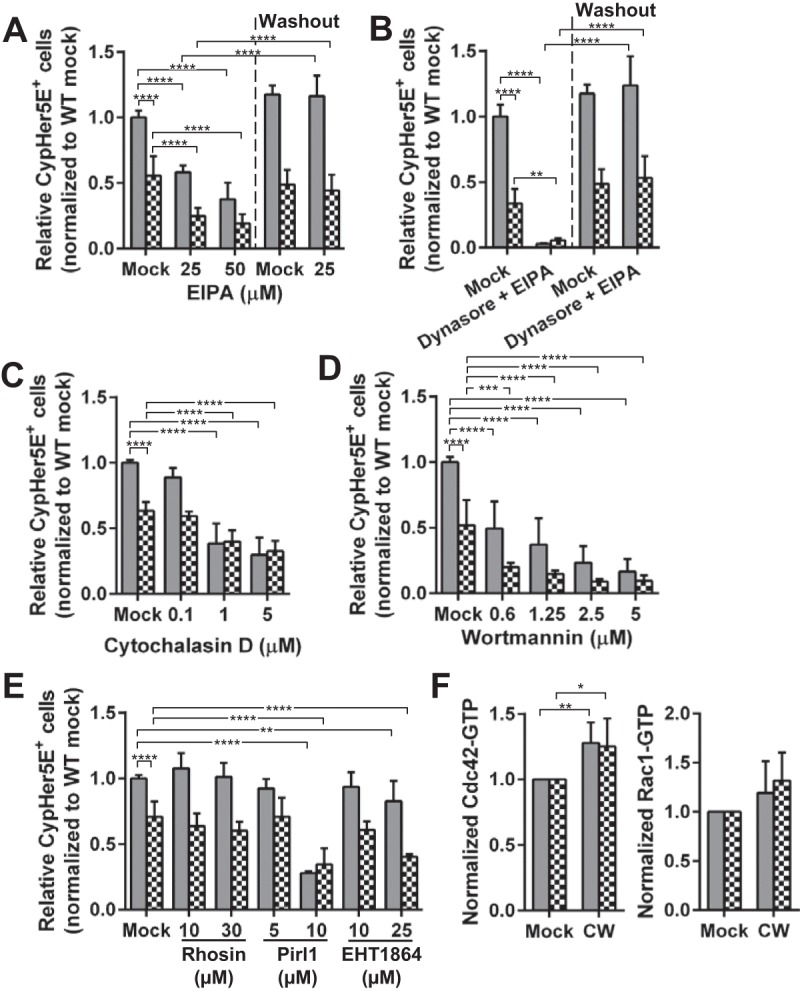
Characteristics of CW uptake independent of PAFr. (A to E) WT MLEC (gray bars) and PAFr KO MLEC (checkerboard bars) were pretreated with EIPA (A), 80 µM dynasore and 25 µM EIPA (B), cytochalasin D (C), wortmannin (D), rhosin (E), Pirl1 (E), and EHT1864 (E) at the indicated concentrations. The cells were incubated with CypHer5E-labeled CW and processed and analyzed as described in the legend to Fig. 1C. Data are presented as means plus SD from three independent experiments. **, *P* < 0.01; ***, *P* < 0.001; ****, *P* < 0.0001 (Bonferroni’s multiple-comparison test). (F) WT MLEC and PAFr KO MLEC were incubated with 10^6^ bacterial equivalents of CW for 1 h at 37°C, incubated immediately on ice, washed once with ice-cold PBS, and lysed and processed as described in Materials and Methods. Quantification of the GTP-bound Cdc42 or Rac1 in the lysates was performed. Data are presented as means plus SD from five (Cdc42) or three (Rac1) independent experiments. *, *P* < 0.05; **, *P* < 0.01 (Bonferroni’s multiple-comparison test).

To further characterize the PAFr/dynamin-independent pathway in WT and PAFr KO MLEC endocytosis of CW, we tested the commonly used inhibitor of macropinocytosis, 5-(*N*-ethyl-*N*-isopropyl) amiloride (EIPA), which is an inhibitor for Na^+^/H^+^ exchangers. The specificity of EIPA was confirmed by the inhibition of pHrodo red dextran uptake without affecting clathrin-dependent transferrin endocytosis (see Fig. S5 in the supplemental material). When WT or PAFr KO MLEC were treated with EIPA as previously described (21–25), CW internalization was inhibited by ~50% in both cell types, suggesting that the PAFr-independent pathway had characteristics of macropinocytosis (Fig. 4A). Cotreatment with 80 µM dynasore and 25 µM EIPA resulted in a strong additive effect, eliminating all CW internalization (Fig. 4B). The observed strong inhibitory effect on CW uptake was not due to cell death (Fig. S7), and the CW uptake was restored when the inhibitors were removed from the medium (Fig. 4A and B).

### Characterization of CW internalization by macropinocytosis.

Macropinocytosis is dependent on actin dynamics, Cdc42, Rac1, and PI3K, but independent of RhoA (26). To investigate these requirements for CW endocytosis, PAFr KO MLEC were treated with various inhibitors. Cytochalasin D (actin depolymerization agent) reduced CW internalization by 40 to 50% compared to that in mock-treated cells (Fig. 4C). Inhibition of PI3K with 0.6 μM wortmannin halved CW internalization by both cell types (Fig. 4D). We also found that CW internalization by PAFr KO MLEC was strongly inhibited by the treatment of Pirl1 (Cdc42 inhibitor) (−50%) and EHT1864 (Rac1 inhibitor) (−40%), but no inhibition was observed in cells treated with rhosin, a RhoA inhibitor (Fig. 4E). These findings were further supported by the detection of increased levels of GTP-bound (activated) Cdc42 and Rac1 in the lysates of cells incubated with CW for 60 min (Fig. 4F). The 60-min incubation time was chosen in these GTPase activation assays, because the GTPases are activated upon the ligand’s trigger and are hydrolyzed to GDP rapidly after internalization is completed.

Although these results demonstrated that the alternative CW endocytosis pathway in the absence of PAFr fulfilled the hallmarks of macropinocytosis (27), enhanced fluid (dextran) uptake, which is characteristic of macropinocytosis, was not observed (see Fig. S6 in the supplemental material). Also, membrane protrusion was not observed in PAFr KO MLEC incubated with CW and stained with wheat germ agglutinin for the plasma membrane (Fig. S4, top panel). However, actin staining showed accumulation of an actin ring at the extracellular CW binding sites (Fig. S4, middle panel, xy and xz view). The line profile of the xy view also showed that the CW signal was surrounded by the actin signal (Fig. S4, bottom panel).

10.1128/mBio.02030-16.4 Figure S4  CW is internalized via an actin-dependent pathway. (A) PAFr KO MLEC was incubated with FITC-labeled CW (green) at 37°C for 45 min, fixed with paraformaldehyde, and immunostained for extracellular CW (magenta) with goat anti-FITC sera without detergent permeabilization, followed by Alexa Fluor 647-conjugated chicken anti-goat sera. Plasma membrane was stained with CF405-conjugated wheat germ agglutinin (WGA). Actin (red) was stained with TRITC-conjugated phalloidin in the presence of saponin. A single confocal slice is shown. Bars, 10 µm. The area enlarged in panel B is shown boxed. Extracellular CW colocalized with actin is indicated with white arrows. (B) An enlarged view of the boxed region in panel A. A xz view of the dotted area is shown. (C) Line profile analysis of the dotted area in panel B is shown. Download Figure S4, EPS file, 6.3 MB. Copyright © 2017 Loh et al. 2017 Loh et al. This content is distributed under the terms of the Creative Commons Attribution 4.0 International license.

10.1128/mBio.02030-16.5 Figure S5  Effects of chemical inhibitors on transferrin and dextran uptake. A549 cells (black bars) or HBMEC (white bars) were pretreated with inhibitors as indicated for 45 min. Transferrin (top) or dextran (bottom) uptake assay was performed as described in Materials and Methods. Data are means ± SD for duplicate samples and are representative of two independent experiments. *, *P* < 0.05; **, *P* < 0.01, ***, *P* < 0.001; ****, *P* < 0.0001 (Bonferroni’s multiple-comparison test). Download Figure S5, EPS file, 2.3 MB. Copyright © 2017 Loh et al. 2017 Loh et al. This content is distributed under the terms of the Creative Commons Attribution 4.0 International license.

10.1128/mBio.02030-16.6 Figure S6  CW does not trigger robust dextran uptake. Flow cytometry analysis of A549 cells and HBMEC incubated for 30 min with fluorescent 40-kDa dextran in the presence or absence of different amounts of CW or with 1 µM phorbol myristate acetate (PMA) as a positive control. Download Figure S6, EPS file, 1.3 MB. Copyright © 2017 Loh et al. 2017 Loh et al. This content is distributed under the terms of the Creative Commons Attribution 4.0 International license.

10.1128/mBio.02030-16.7 Figure S7  Effects of chemical inhibitors on cell viability. WT MLEC (gray bars) or PAFr KO MLEC (checkerboard bars) treated with chemical inhibitors as indicated and assessed for CW uptake were gated for live cells either by excluding low forward scatter (FSC^lo^) or high DAPI (DAPI^hi^) or by excluding FSC^lo^, high SSC (SSC^hi^) populations. Data are means ± SD of at least two independent experiments performed in triplicate. *, *P* < 0.05; **, *P* < 0.01; ****, *P* < 0.0001 (Bonferroni’s multiple-comparison test). Download Figure S7, EPS file, 2.7 MB. Copyright © 2017 Loh et al. 2017 Loh et al. This content is distributed under the terms of the Creative Commons Attribution 4.0 International license.

Taken together, the data suggested that the alternative CW uptake pathway resembled macropinocytosis; however, the lack of enhanced fluid uptake and membrane ruffles, which are key features of macropinocytosis, distinguished it from the classical macropinocytosis pathway. This alternative pathway shares similarity with the human papillomavirus type 16 (HPV-16) endocytosis, which is also sensitive to EIPA and dependent on actin dynamics (28). Therefore, we concluded that the PAFr-independent pathway is independent of clathrin, caveolin 1, and dynamin but requires actin dynamics, Na^+^/H^+^ exchangers, PI3K, Cdc42, and Rac1.

### Intracellular trafficking of CW and signaling from endosomes.

**FIG 5:**
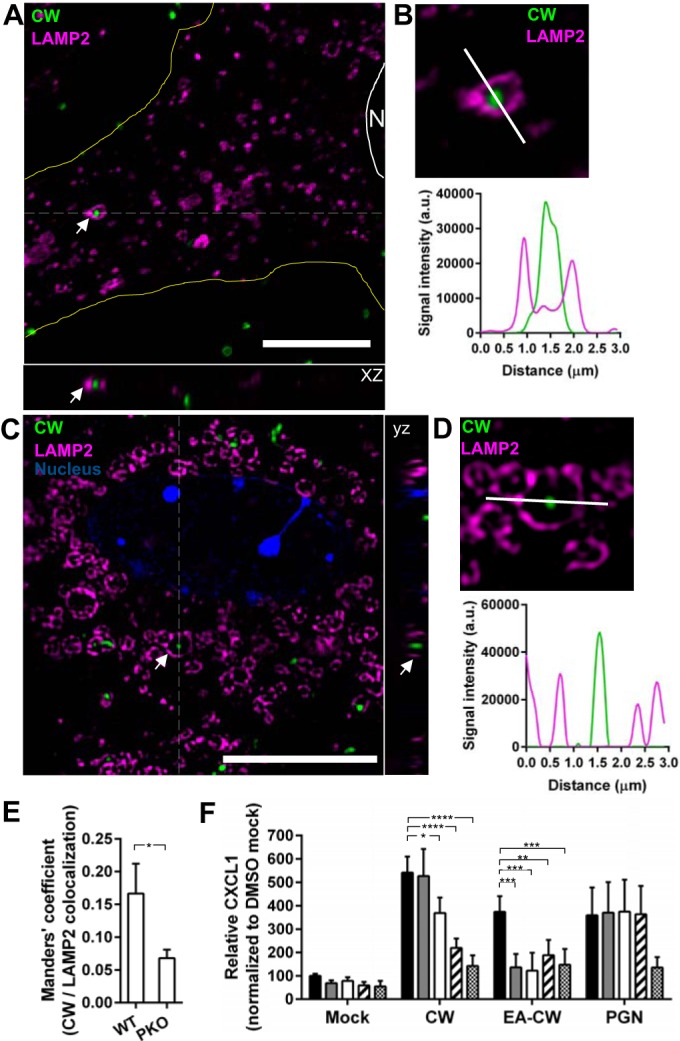
CW trafficking to lysosomes. (A) WT MLEC were incubated with FITC-labeled CW (green) for 2 h at 37°C, fixed with methanol, and immunostained for LAMP2 (magenta). A representative deconvolved high-magnification single confocal slice is shown. The white scale bar represents 10 µm. The cell periphery was outlined in yellow. The white line and N indicate the cell nucleus. An example of CW localized in a LAMP2-positive endosome is indicated by the white arrows. An xz view of the dotted area is shown. (B, top) An enlarged view of the same CW-containing LAMP2 endosome in panel A is shown. The white line indicates a line profile analysis of the deconvolved confocal image. (Bottom) The values for each signal were plotted. a.u., arbitrary units. (C) WT MLEC were incubated with CF640-labeled CW (green) and stained for LAMP2 (magenta) as described above for panel A. Images were acquired with SIM. A single z plane of an enlarged image of a cell is shown. The white scale bar represents 10 µm. The nucleus (blue) was stained with DAPI. Examples of CW localized in LAMP2-positive endosome are indicated with white arrows. A yz view of the dotted area is shown. (D, top) An enlarged view of the same CW-containing LAMP2 endosome in panel C) is shown. The white line indicates a line profile analysis of the SIM image. (Bottom) The values for each signal were plotted. (E) Quantification of Manders’ coefficient for CW colocalized with LAMP2 in WT MLEC and PAFr KO MLEC after 2-h incubation. At least six random fields with 70 to 100 cells were quantified. Data are means plus standard errors of the means from three independent experiments. *, *P* < 0.05 (Student’s *t* test). (F) WT MLEC were pretreated with DMSO (black bars), 0.1 µM bafilomycin A1 (gray bars), 0.5 µM bafilomycin A1 (white bars), 1 µM cytochalasin D (hatched bars), or 50 µM EIPA (checkerboard bars) and then exposed to CW, EA-CW, or staphylococcal peptidoglycan (PGN) for 8 h at 37°C. CXCL1 in the medium was analyzed by ELISA. Data are means plus SD from three independent experiments performed in duplicate. *, *P* < 0.05; **, *P* < 0.01; ***, *P* < 0.001; ****, *P* < 0.0001 (Bonferroni’s multiple-comparison test).

Following internalization into WT and PAFr KO cells, CW was found to localize in compartments positive for lysosome-associated membrane glycoprotein 2 (LAMP2), which indicated trafficking to the late/lysosomal compartment (Fig. 5A and B). Structural illumination microscopy (SIM) revealed that the size of the CW-containing LAMP2 compartment was >1 µm (Fig. 5C and D). Colocalization studies, which quantified the Manders’ coefficient for the fraction of CW overlap with LAMP2 from at least six random fields with 70 to 100 cells, showed that more CW colocalized with LAMP2 in WT MLEC than in PAFr KO MLEC (Fig. 5E). These data agreed with the flow cytometry data that CW uptake was occurring by two pathways (Fig. 1D).

Intriguingly, we found that treatment of WT cells with bafilomycin A1, which is a lysosomal inhibitor, reduced CW-induced CXCL1 significantly, and the effect was even more drastic for EA-CW-treated cells, but no reduction was observed in staphylococcal peptidoglycan (PGN)-treated cells (Fig. 5F). We also observed significant reduction in CW- or EA-CW-induced CXCL1 in cells treated with cytochalasin D and EIPA, but PGN-induced CXCL1 was unaffected by cytochalasin D. Taken together with the complete absence of signaling in the TLR2-deficient cells, our findings indicate that CW internalization via the actin-dependent pathway might have a role in the production of proinflammatory cytokines from the lysosomal compartment in a TLR2-dependent manner.

## DISCUSSION

**FIG 6:**
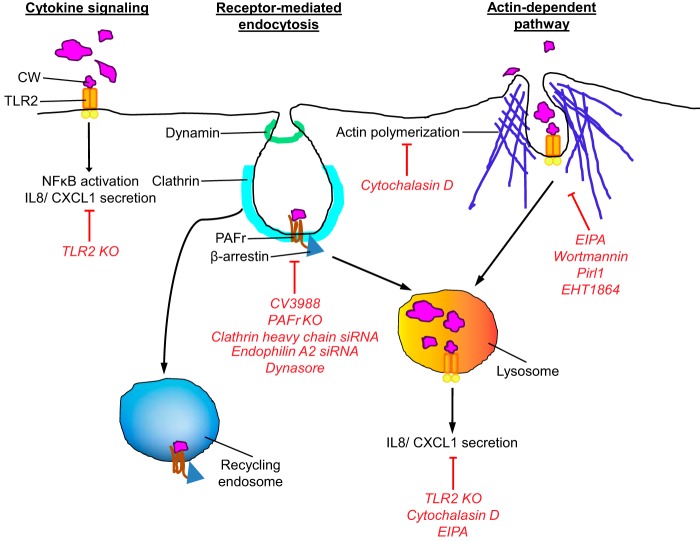
Current model of CW entry and signaling. Schematic representations of three pathways are shown. Red lines and text indicate inhibition by listed treatments. (Left) The CW-TLR2 interaction is essential for activating NF-κB and proinflammatory cytokine secretion, but not for CW internalization. (Middle) On the other hand, the CW-PAFr interaction is required for CW internalization via the clathrin/dynamin-dependent pathway but is not essential for inflammatory responses. Upon internalization via PAFr, CW-containing endosomes are trafficked either to recycling endosomes or lysosomes. (Right) An additional EIPA-sensitive actin-dependent pathway is involved in CW internalization leading to the lysosomal compartment. Some of the signals for TLR2 CW-induced proinflammatory cytokines originated from lysosomal compartments.

The results of this study indicate that Gram-positive CW interacts with at least three pathways with different fates (Fig. 6). For receptor-mediated events recognizing the CW at the cell membrane, new data clarified that ligation of TLR2 did not lead to CW uptake but strongly activated inflammatory cytokine signaling. In contrast, the PAFr uptake pathway, shared by pathogens that bear PCho, accounted for ~40% of CW uptake, involved dynamin and clathrin, but not caveolin, and targeted CW to a vesicle with no inflammatory signaling (29).

CW internalization was not abolished in PAFr-deficient cells, indicating that there is an additional uptake pathway. The alternative pathway accounted for approximately ~50% of CW uptake and was sensitive to EIPA and dependent on actin dynamics. Phosphorylcholine-deficient EA-CW appeared to be the best ligand to exclusively engage this pathway. Uptake into a vesicle was independent of dynamin, clathrin, and caveolin and trafficked CW-containing endosomes to the lysosome. Staphylococcal PGN uptake might share features with this pathway but lacked involvement of actin. TLR2 appeared to be involved in signaling activation of cytokine production by CW from the endosome.

The PAFr-independent pathway was sensitive to EIPA. Macropinocytosis has been described as a mode of host cell invasion for some bacteria, such as *Salmonella enterica* serotype Typhimurium, and *Haemophilus influenzae*, which inject a cocktail of the bacterial proteins into the cytosol of nonphagocytic cells, resulting in actin rearrangement and membrane ruffle formation (30, 31). In recent years, viruses, such as influenza virus, respiratory syncytial virus, Ebola virus, human papillomavirus type 16 (HPV-16), vaccinia virus, etc., have also been demonstrated to induce macropinocytosis for viral entry *in vitro* (23, 32–36). These viruses mimic apoptotic cells by exposing phosphatidylserine on the external virion surface, and the engagement of the viruses on phosphatidylserine receptors (such as T cell immunoglobulin mucin [TIM] and Tyro3, Axl, and Mer [TAM] receptors) triggers macropinocytosis (37). Unlike intact bacteria and viruses, our purified CW pieces are fragments of pneumococcal peptidoglycan and teichoic acid complexes and do not contain phosphatidylserine (evidenced by the absence of annexin V staining [data not shown]). Although sensitive to the Na^+^/H^+^ exchanger inhibitor EIPA, the PAFr-independent pathway did not enhance fluid uptake, and no membrane ruffles or blebs were observed; hence, it appears to be different from classical macropinocytosis. This alternative pathway more closely resembles the HPV-16-induced internalization pathway, which is also sensitive to EIPA and dependent on actin dynamics and tyrosine kinase signaling but lacks membrane ruffles or blebs (28). The virus was found in small coat-free plasma membrane invaginations of 65 to 120 nm in diameter (28). Although EIPA sensitivity has been listed as one of the main criteria for macropinocytosis (26, 27), the inhibitor’s mechanism of action remains elusive (38). Recently, EIPA has also been shown to inhibit the uptake of cholera toxin B, which is endocytosed via clathrin-independent carriers (CLIC) and caveolae pathways (25). The newly described FEME pathway is also sensitive to EIPA (17). Therefore, EIPA sensitivity should not be considered a defining feature for macropinocytosis, and it is possible that there are other novel non-macropinocytic pathways that are sensitive to EIPA.

Recently, Irving et al. reported that the cytoplasmic pathogen recognition receptor (PRR) nucleotide-binding oligomerization domain 1 (Nod1) was recruited to Gram-negative bacterial peptidoglycan-containing endosomes, which promoted receptor-interacting protein 2 (RIP2)-dependent autophagy and inflammatory responses of epithelial cells to infection (39). We found the majority of the internalized Gram-positive CW was in endosomes positive for LAMP2, but devoid of Nod2 receptor, which senses peptidoglycan (40). Previous findings showed that CW-induced inflammatory cytokine production by macrophages was TLR2 dependent but Nod2 independent, while the CW-induced anti-inflammatory cytokine IL-10 production was dependent on Nod2 and receptor-interacting serine/threonine protein kinase 2 (RIPK2) (41). In this study, TLR2 KO endothelial cells with intact Nod2 receptor failed to induce either inflammatory cytokines or IL-10 secretion (Fig. S1). The discrepancy between both studies could be due to the difference in cell types, and it has been reported that endothelial cells do not produce IL-10 (42). Alternatively, the detection of ligand, such as muramyl dipeptide (MDP), by Nod2 receptor occurs in the cytosol, and the size of the CW complex might be restricting it from crossing the lysosomal membrane via the endolysosomal peptide transporters (43). Further, Davis et al. demonstrated that the sensing of peptidoglycan by nucleotide-binding oligomerization domain (NOD) required pneumolysin for puncturing the endosomal membrane of macrophages (11), which further suggested that CW alone is insufficient to cross the endosomal membrane.

Our data suggested that lysosomes have a role in CW-induced inflammatory response. We observed that CW-induced CXCL1 production was reduced in cells treated with bafilomycin A1, and the impact was more drastic in EA-CW-treated cells. Combined with the complete abolition of signaling in TLR2 KO cells, these data suggested that the lysosomal compartment can have a role in host innate defense by detecting internalized bacterial CW complexes in a TLR2-dependent manner. TLR2 enrichment has been found in yeast CW particle zymosan-containing phagosomes (46), and receptor signaling from endosomes has been suggested by Brandt et al. and Stack et al. (44–45). Our data also raise a rationale for the pneumococcus to decorate its surface with PCho and to hijack PAFr for cell entry, which would minimize immune cell activation and thus promote infection (Fig. 5F).

The basic structure of pneumococcal CW is conserved, but variations exist at the stem peptide composition, the interpeptide crossbridge, and in the mode of cross-linkage (47, 48). The variations in the stem peptide composition might affect the binding of the CW to TLR2, which might result in a difference in the degree of inflammation (49, 50). However, the uptake of CW by both pathways is independent of TLR2 (Fig. 1D); hence, a similar uptake pathway would be predicted for CW from other *Streptococcus pneumoniae* strains regardless of the stem peptide composition.

In summary, we used purified pneumococcal CW as a model of Gram-positive peptidoglycan-teichoic acid complex to study its interactions with epithelial and endothelial cells. Three interactions were identified (Fig. 6). CW engagement of TLR2 resulted in inflammatory signaling without uptake into cells. Two uptake pathways were distinguished. CW internalized by PAFr was dependent on dynamin and clathrin and induced no inflammatory signaling. This is consistent with evidence that CW traffics across barriers via PAFr (1, 51), perhaps minimizing cellular activation so as to avoid clearance by immune cells. A second new pathway of uptake had features of macropinocytosis and appeared to traffic CW to lysosomes accompanied by cytokine production.

## MATERIALS AND METHODS

### Cell cultures and mouse lung endothelial cells.

A549 (ATCC, Manassas, VA) lung epithelial cells were maintained in Dulbecco modified Eagle medium (DMEM) (Sigma-Aldrich, St. Louis, MO) supplemented with 10% (vol/vol) heat-inactivated fetal bovine serum (FBS) (Atlanta Biologicals, Lawrenceville, GA), and GlutaMAX (Invitrogen). Primary human brain microvascular endothelial cells (HBMEC) (ScienCell Research Laboratories, Carlsbad, CA) were maintained in endothelial cell medium (ECM) (ScienCell Research Laboratories, Carlsbad, CA) supplemented with 5% (vol/vol) heat-inactivated FBS, and endothelial cell growth supplement (ECGS) as described in the manufacturer’s instructions.

Mouse lung endothelial cells (MLEC) were isolated from 4- to 6-week-old C57BL/6 mice by the method of Fehrenbach et al. (52). The sorted intercellular adhesion molecule 2 (ICAM-2)-positive cells were maintained in MLEC complete growth medium containing 1:1 low-glucose DMEM (Sigma-Aldrich, St. Louis, MO), and Ham’s F-12 (Sigma-Aldrich, St. Louis, MO) supplemented with 20% (vol/vol) FBS, 0.2 U/ml heparin, 1× Glutamax, 0.1 mg/ml ECGS (Alfa Aesar, Ward Hill, MA), and penicillin-streptomycin.

All experiments involving animals were performed with prior approval of and in accordance with guidelines of the St. Jude Institutional Animal Care and Use Committee (protocol 250-100332-12/14). The St. Jude laboratory animal facilities have been fully accredited by the American Association for Accreditation of Laboratory Animal Care. Laboratory animals are maintained in accordance with the applicable portions of the Animal Welfare Act and the guidelines prescribed in the *Guide for the Care and Use of Laboratory* *Animals* (53).

### CW preparation.

*Streptococcus pneumoniae* R6 strain was grown in media containing choline or adapted to ethanolamine as described previously (54). CW purification was performed as described previously (1, 5). The CW product contained teichoic acid linked to peptidoglycan but was devoid of proteins, lipoteichoic acids, and noncovalent adducts (5, 55). The level of contaminating endotoxin was assessed to be below detection by Pierce LAL chromogenic endotoxin quantitation kit (Thermo Scientific). The lyophilized CW was resuspended in endotoxin-free water to the concentration of 10^6^ bacterial equivalents/µl and stored at 4°C. CW was labeled with 0.1 mg/ml CypHer5E *N*-hydroxysuccinimide (NHS) ester (GE Healthcare) or with 0.1 mg/ml fluorescein isothiocyanate (FITC) or 0.05 mg/ml CF488 or CF640 NHS ester (Biotium, Hayward, CA) in carbonate buffer (pH 9.2) for 2 h or 1 h, respectively, at room temperature, protected from light, and then washed three times with plain medium.

### Chemokine ELISA and cytokine array.

The cells were incubated with CW at 37°C for 8 h or 24 h, and the culture medium was collected. The amount of CXCL1 in culture medium was determined using the Mouse CXCL1/KC DuoSet ELISA (R&D Systems, Minneapolis, MN) or mouse inflammation array C1 membrane (RayBiotech) according to the manufacturer’s instruction.

### CW endocytosis assays.

A full list of antibodies, inhibitors, and siRNAs used appears in Table S1 in the supplemental material.

10.1128/mBio.02030-16.8 Table S1  List of the antibodies, pharmacological inhibitors, and siRNAs that were used in this study. Download Table S1, DOCX file, 0.02 MB. Copyright © 2017 Loh et al. 2017 Loh et al. This content is distributed under the terms of the Creative Commons Attribution 4.0 International license.

For siRNA-mediated knockdown experiments, cells were plated in a 24-well plate and A549 cells were transfected with 50 nM siRNA oligonucleotides by using Lipofectamine RNAiMAX (Invitrogen) when the cell density achieved 70 to 80% confluence. The transfection was repeated 24 h after the first transfection, and cells were used for assay 24 h after the second round of transfection. For MLEC, a single dose of siRNA transfection was performed with Lipofectamine RNAiMAX, as described for A549 cells, and used for assay 36 h after the transfection. For primary HBMEC, cells were plated in a fibronectin-coated 24-well plate, transfected with 25 nM siRNA oligonucleotides by using DharmaFECT 1 (Thermo Scientific), and cells were used for assay 48 h after the transfection. Protein depletion was determined by immunoblotting.

For chemical inhibitor studies, cells were treated with drugs at indicated concentration for 45 min in medium containing 10% (vol/vol) heat-inactivated NuSerum (56). Labeled CW (10^6^ bacterial equivalents) was added to cells, centrifuged at 700 × *g* and 4°C for 5 min, and incubated at 37°C for 2 h. CW uptake was stopped by washing three times with cold phosphate-buffered saline (PBS); cells were detached by TrypLE Express (Invitrogen), resuspended in cold 2% FBS in PBS (fluorescence-activated cell sorting [FACS] buffer), and fluorescence was analyzed using BD LSR Fortessa and FlowJo software (Treestar). At least 10,000 cells were counted for each sample. Cytotoxicity was assessed by the incorporation of 4′,6′-diamidino-2-phenylindole (DAPI) (Biotium, Hayward, CA).

For inhibitor washout assay, inhibitor-treated cells were washed three times with prewarmed medium without FBS, replenished with medium, and incubated at 37°C and 5% CO_2_ for 2 h before adding CypHer5E NHS ester-labeled CW (CypHer5E-labeled CW). CW endocytosis assay was performed as described above.

Positive controls for inhibitor studies included transferrin and dextran uptake assays. The cells were incubated on ice with 25 µg/ml Alexa Fluor 647- or tetramethylrhodamine (TRITC)-conjugated transferrin (Molecular Probes) in plain medium for 20 min. The cells were then washed with cold plain medium and transferred to 37°C for 15 min. Endocytosis was stopped by ice-cold PBS washes, and surface-bound transferrin was removed by acid wash for 2 min on ice in 150 mM glycine, pH 3.0. The cells were then processed for flow cytometry analysis. Alternatively, cells were incubated on ice with 50 µg/ml pHrodo red dextran with a molecular weight (MW) of 10,000 (Molecular Probes) and transferred to 37°C for 20 min. Endocytosis was stopped by incubating the plate on ice and washing the cells three times with ice-cold PBS. The cells were processed for flow cytometry analysis.

### Immunofluorescence and confocal microscopy imaging.

The cells were plated on glass coverslips (neuVitro, Vancouver, WA) in a 24-well tissue culture plate and incubated with fluorescent dye-labeled CW. The cells were fixed with 3% paraformaldehyde (EMS, Hatfield, PA) dissolved in PHEM buffer [25 mM HEPES, 10 mM EGTA, 60 mM piperazine-*N*,*N*′-bis(2-ethanesulfonic acid) (PIPES), 2 mM MgCl_2_] for 20 min at room temperature. For methanol fixation, cells were fixed with anhydrous methanol for 10 min at −20°C. The cells were then washed three times for 5 min each time with wash solution (PBS containing 5% [vol/vol] FBS). The cellular structures in the cells were stained with primary antibody (see Table S1 in the supplemental material) at 4°C overnight. When unlabeled primary antibody was used, cells were stained with fluorescent dye-labeled secondary antibodies at room temperature for 1 h. Actin was stained with 0.025 mg/ml TRITC-conjugated phalloidin (Sigma-Aldrich, St. Louis, MO), and plasma membrane was stained with 5 µg/ml CF405M-conjugated wheat germ agglutinin (WGA) (Biotium, Hayward, CA). For differential staining of intra- and extracellular CW, cells were stained with anti-FITC sera, followed by Alexa Fluor 647-conjugated chicken anti-goat IgG sera without saponin permeabilization. The nuclei of the cells were stained with 1 µg/ml DAPI (Biotium, Hayward, CA), and cells were mounted in Confocal Matrix (Micro Tech Lab, Graz, Austria). Confocal images were acquired with LSM780 confocal microscope (Carl Zeiss MicroImaging). All confocal images represent a single plane acquired with a 63× plan-Apochromat oil immersion objective with a numerical aperture (NA) of 1.40 with the confocal pinhole set at one Airy unit. Structured illumination microscopy (SIM) images were acquired in Zeiss Elyra PS.1 and processed by using Zeiss acquisition software ZEN. Multilocation z-stack images for colocalization study was acquired using a Marianas confocal microscope system (Intelligent Imaging Innovations, Denver, CO), which incorporates a Zeiss Axioplan microscope equipped with a Definite Focus system (Carl Zeiss, Inc.), a Yokogawa CSUX spinning disk confocal scan head, and a Photometrics Evolve charge-coupled-device (CCD) camera (Photometrics, Tucson, AZ). Excitation was by diode lasers at 488 and 642 nm. z-stack confocal images were acquired with a 63× 1.4-NA (oil immersion) plan-Apochromat objective using Slidebook 6 software (Intelligent Imaging Innovations). Colocalization analyses were performed on at least six random fields with 70 to 100 cells and in three independent experiments using ImageJ plugin Intensity Correlation Analysis. Image deconvolution was performed with a blind deconvolution algorithm for aberration correction and refractive index 1.41 with Autoquant X3 (Media Cybernetics, Rockville, MD).

### Rho family GTPase activation assays.

Activation of Rho family GTPases was quantified by colorimetric G-LISA GTPase Activation Assay Biochem kit (Cytoskeleton, Inc., Denver, CO) according to the manufacturer’s instructions. Briefly, MLEC were cultured to confluence in fibronectin-coated 24-well tissue culture plates. The cells were serum starved for at least 5 h. The cells were incubated with 10^6^ bacterial equivalents of CW for 1 h at 37°C with 5% CO_2_. At the end of the incubation, cells were incubated immediately in ice, washed once with ice-cold PBS, and lysed in ice-cold lysis buffer supplemented with protease inhibitor. The lysate was clarified at 10,000 × *g* at 4°C for 1 min. An aliquot (20 µl) of the lysate was used for protein concentration quantification by using Precision Red Advanced Protein assay provided with the kit. The remaining lysate was snap-frozen in liquid nitrogen and stored at −70°C. Snap-frozen lysate was then thawed, and the protein concentrations were equalized with ice-cold lysis buffer. The activated GTPases were determined per the manufacturer’s instructions. The absorbance of the horseradish peroxidase (HRP) signal was read at 490 nm with a Spectra MAX 340 microplate reader (Molecular Device).

### Statistical analysis.

All experiments were performed in triplicate and repeated at least twice. Data were analyzed with GraphPad Prism version 6.05. For data from two experimental conditions compared with each other, two-tailed Student’s *t* test was used to test statistical significance. In instances where multiple experimental conditions were compared with a single control group, statistical significance was tested using one-way analysis of variance (ANOVA) followed by Bonferroni’s multiple-comparison postcomparison test. A *P* value less than 0.05 was considered significant.

## References

[1] Fillon S, Soulis K, Rajasekaran S, Benedict-Hamilton H, Radin JN, Orihuela CJ, El Kasmi KC, Murti G, Kaushal D, Gaber MW, Weber JR, Murray PJ, Tuomanen EI 2006 Platelet-activating factor receptor and innate immunity: uptake of Gram-positive bacterial cell wall into host cells and cell-specific pathophysiology. J Immunol 177:6182–6191. doi:10.4049/jimmunol.177.9.6182. 17056547

[2] Orihuela CJ, Fillon S, Smith-Sielicki SH, El Kasmi KC, Gao G, Soulis K, Patil A, Murray PJ, Tuomanen EI 2006 Cell wall-mediated neuronal damage in early sepsis. Infect Immun 74:3783–3789. doi:10.1128/IAI.00022-06. 16790750 PMC1489725

[3] Clarke TB, Davis KM, Lysenko ES, Zhou AY, Yu Y, Weiser JN 2010 Recognition of peptidoglycan from the microbiota by Nod1 enhances systemic innate immunity. Nat Med 16:228–231. doi:10.1038/nm.2087. 20081863 PMC4497535

[4] Schneider O, Michel U, Zysk G, Dubuis O, Nau R 1999 Clinical outcome in pneumococcal meningitis correlates with CSF lipoteichoic acid concentrations. Neurology 53:1584–1587. doi:10.1212/WNL.53.7.1584. 10534274

[5] Tuomanen E, Liu H, Hengstler B, Zak O, Tomasz A 1985 The induction of meningeal inflammation by components of the pneumococcal cell wall. J Infect Dis 151:859–868. doi:10.1093/infdis/151.5.859. 3989321

[6] Tuomanen E, Rich R, Zak O 1987 Induction of pulmonary inflammation by components of the pneumococcal cell surface. Am Rev Respir Dis 135:869–874. doi:10.1164/arrd.1987.135.4.869. 3565933

[7] Yoshimura A, Lien E, Ingalls RR, Tuomanen E, Dziarski R, Golenbock D 1999 Recognition of Gram-positive bacterial cell wall components by the innate immune system occurs via Toll-like receptor 2. J Immunol 163:1–5. 10384090

[8] Tsuchiya K, Toyama K, Tsuprun V, Hamajima Y, Kim Y, Ondrey FG, Lin J 2007 Pneumococcal peptidoglycan-polysaccharides induce the expression of interleukin-8 in airway epithelial cells by way of nuclear factor-kappaB, nuclear factor interleukin-6, or activation protein-1 dependent mechanisms. Laryngoscope 117:86–91. doi:10.1097/01.mlg.0000244182.81768.31. 17135982 PMC2847848

[9] Schumann RR, Pfeil D, Freyer D, Buerger W, Lamping N, Kirschning CJ, Goebel UB, Weber JR 1998 Lipopolysaccharide and pneumococcal cell wall components activate the mitogen activated protein kinases (MAPK) erk-1, erk-2, and p38 in astrocytes. Glia 22:295–305. doi:10.1002/(SICI)1098-1136(199803)22:3<295::AID-GLIA8>3.0.CO;2-4. 9482215

[10] Spellerberg B, Rosenow C, Sha W, Tuomanen EI 1996 Pneumococcal cell wall activates NF-κB in human monocytes: aspects distinct from endotoxin. Microb Pathog 20:309–317. doi:10.1006/mpat.1996.0029. 8861395

[11] Davis KM, Nakamura S, Weiser JN 2011 Nod2 sensing of lysozyme-digested peptidoglycan promotes macrophage recruitment and clearance of *S. pneumoniae* colonization in mice. J Clin Invest 121:3666–3676. doi:10.1172/JCI57761. 21841315 PMC3163965

[12] Cundell DR, Gerard NP, Gerard C, Idanpaan-Heikkila I, Tuomanen EI 1995 *Streptococcus pneumoniae* anchor to activated human cells by the receptor for platelet-activating factor. Nature 377:435–438. doi:10.1038/377435a0. 7566121

[13] Rijneveld AW, Weijer S, Florquin S, Speelman P, Shimizu T, Ishii S, van der Poll T 2004 Improved host defense against pneumococcal pneumonia in platelet-activating factor receptor-deficient mice. J Infect Dis 189:711–716. doi:10.1086/381392. 14767826

[14] Ring A, Weiser JN, Tuomanen EI 1998 Pneumococcal trafficking across the blood-brain barrier. Molecular analysis of a novel bidirectional pathway. J Clin Invest 102:347–360. doi:10.1172/JCI2406. 9664076 PMC508893

[15] Eckels PC, Banerjee A, Moore EE, McLaughlin NJ, Gries LM, Kelher MR, England KM, Gamboni-Robertson F, Khan SY, Silliman CC 2009 Amantadine inhibits platelet-activating factor induced clathrin-mediated endocytosis in human neutrophils. Am J Physiol Cell Physiol 297:C886–C897. doi:10.1152/ajpcell.00416.2008. 19295175 PMC2770739

[16] McLaughlin NJ, Banerjee A, Kelher MR, Gamboni-Robertson F, Hamiel C, Sheppard FR, Moore EE, Silliman CC 2006 Platelet-activating factor-induced clathrin-mediated endocytosis requires β-arrestin-1 recruitment and activation of the p38 MAPK signalosome at the plasma membrane for actin bundle formation. J Immunol 176:7039–7050. doi:10.4049/jimmunol.176.11.7039. 16709866

[17] Boucrot E, Ferreira AP, Almeida-Souza L, Debard S, Vallis Y, Howard G, Bertot L, Sauvonnet N, McMahon HT 2015 Endophilin marks and controls a clathrin-independent endocytic pathway. Nature 517:460–465. doi:10.1038/nature14067. 25517094

[18] Iovino F, Molema G, Bijlsma JJ 2014 *Streptococcus pneumoniae* interacts with pIgR expressed by the brain microvascular endothelium but does not co-localize with PAF receptor. PLoS One 9:e97914. doi:10.1371/journal.pone.0097914. 24841255 PMC4026408

[19] Gradstedt H, Iovino F, Bijlsma JJ 2013 *Streptococcus pneumoniae* invades endothelial host cells via multiple pathways and is killed in a lysosome dependent manner. PLoS One 8:e65626. doi:10.1371/journal.pone.0065626. 23785439 PMC3681976

[20] Asmat TM, Agarwal V, Saleh M, Hammerschmidt S 2014 Endocytosis of *Streptococcus pneumoniae* via the polymeric immunoglobulin receptor of epithelial cells relies on clathrin and caveolin dependent mechanisms. Int J Med Microbiol 304:1233–1246. doi:10.1016/j.ijmm.2014.10.001. 25455218

[21] Holla P, Ahmad I, Ahmed Z, Jameel S 2015 Hepatitis E virus enters liver cells through a dynamin-2, clathrin and membrane cholesterol-dependent pathway. Traffic 16:398–416. doi:10.1111/tra.12260. 25615268

[22] Hetzenecker S, Helenius A, Krzyzaniak MA 2016 HCMV induces macropinocytosis for host cell entry in fibroblasts. Traffic 17:351–368. doi:10.1111/tra.12355. 26650385

[23] Krzyzaniak MA, Zumstein MT, Gerez JA, Picotti P, Helenius A 2013 Host cell entry of respiratory syncytial virus involves macropinocytosis followed by proteolytic activation of the F protein. PLoS Pathog 9:e1003309. doi:10.1371/journal.ppat.1003309. 23593008 PMC3623752

[24] Krieger SE, Kim C, Zhang L, Marjomaki V, Bergelson JM 2013 Echovirus 1 entry into polarized Caco-2 cells depends on dynamin, cholesterol, and cellular factors associated with macropinocytosis. J Virol 87:8884–8895. doi:10.1128/JVI.03415-12. 23740983 PMC3754084

[25] Nonnenmacher M, Weber T 2011 Adeno-associated virus 2 infection requires endocytosis through the CLIC/GEEC pathway. Cell Host Microbe 10:563–576. doi:10.1016/j.chom.2011.10.014. 22177561 PMC3257174

[26] Mercer J, Helenius A 2009 Virus entry by macropinocytosis. Nat Cell Biol 11:510–520. doi:10.1038/ncb0509-510. 19404330

[27] Mercer J, Helenius A 2012 Gulping rather than sipping: macropinocytosis as a way of virus entry. Curr Opin Microbiol 15:490–499. doi:10.1016/j.mib.2012.05.016. 22749376

[28] Schelhaas M, Shah B, Holzer M, Blattmann P, Kühling L, Day PM, Schiller JT, Helenius A 2012 Entry of human papillomavirus type 16 by actin-dependent, clathrin- and lipid raft-independent endocytosis. PLoS Pathog 8:e1002657. doi:10.1371/journal.ppat.1002657. 22536154 PMC3334892

[29] García Rodríguez C, Cundell DR, Tuomanen EI, Kolakowski LF Jr, Gerard C, Gerard NP 1995 The role of N-glycosylation for functional expression of the human platelet-activating factor receptor. Glycosylation is required for efficient membrane trafficking. J Biol Chem 270:25178–25184. doi:10.1074/jbc.270.42.25178. 7559653

[30] Ketterer MR, Shao JQ, Hornick DB, Buscher B, Bandi VK, Apicella MA 1999 Infection of primary human bronchial epithelial cells by *Haemophilus influenzae*: macropinocytosis as a mechanism of airway epithelial cell entry. Infect Immun 67:4161–4170. 10417188 10.1128/iai.67.8.4161-4170.1999 PMC96721

[31] Garcia-del Portillo F, Finlay BB 1994 *Salmonella* invasion of nonphagocytic cells induces formation of macropinosomes in the host cell. Infect Immun 62:4641–4645. 7927733 10.1128/iai.62.10.4641-4645.1994 PMC303156

[32] Aleksandrowicz P, Marzi A, Biedenkopf N, Beimforde N, Becker S, Hoenen T, Feldmann H, Schnittler HJ 2011 Ebola virus enters host cells by macropinocytosis and clathrin-mediated endocytosis. J Infect Dis 204(Suppl 3):S957–S967. doi:10.1093/infdis/jir326. 21987776 PMC3189988

[33] de Vries E, Tscherne DM, Wienholts MJ, Cobos-Jiménez V, Scholte F, García-Sastre A, Rottier PJ, de Haan CA 2011 Dissection of the influenza A virus endocytic routes reveals macropinocytosis as an alternative entry pathway. PLoS Pathog 7:e1001329. doi:10.1371/journal.ppat.1001329. 21483486 PMC3068995

[34] Mulherkar N, Raaben M, de la Torre JC, Whelan SP, Chandran K 2011 The Ebola virus glycoprotein mediates entry via a non-classical dynamin-dependent macropinocytic pathway. Virology 419:72–83. doi:10.1016/j.virol.2011.08.009. 21907381 PMC3177976

[35] Nanbo A, Imai M, Watanabe S, Noda T, Takahashi K, Neumann G, Halfmann P, Kawaoka Y 2010 Ebolavirus is internalized into host cells via macropinocytosis in a viral glycoprotein-dependent manner. PLoS Pathog 6:e1001121. doi:10.1371/journal.ppat.1001121. 20886108 PMC2944813

[36] Sandgren KJ, Wilkinson J, Miranda-Saksena M, McInerney GM, Byth-Wilson K, Robinson PJ, Cunningham AL 2010 A differential role for macropinocytosis in mediating entry of the two forms of vaccinia virus into dendritic cells. PLoS Pathog 6:e1000866. doi:10.1371/journal.ppat.1000866. 20421949 PMC2858709

[37] Amara A, Mercer J 2015 Viral apoptotic mimicry. Nat Rev Microbiol 13:461–469. doi:10.1038/nrmicro3469. 26052667 PMC7097103

[38] Koivusalo M, Welch C, Hayashi H, Scott CC, Kim M, Alexander T, Touret N, Hahn KM, Grinstein S 2010 Amiloride inhibits macropinocytosis by lowering submembranous pH and preventing Rac1 and Cdc42 signaling. J Cell Biol 188:547–563. doi:10.1083/jcb.200908086. 20156964 PMC2828922

[39] Irving AT, Mimuro H, Kufer TA, Lo C, Wheeler R, Turner LJ, Thomas BJ, Malosse C, Gantier MP, Casillas LN, Votta BJ, Bertin J, Boneca IG, Sasakawa C, Philpott DJ, Ferrero RL, Kaparakis-Liaskos M 2014 The immune receptor NOD1 and kinase RIP2 interact with bacterial peptidoglycan on early endosomes to promote autophagy and inflammatory signaling. Cell Host Microbe 15:623–635. doi:10.1016/j.chom.2014.04.001. 24746552

[40] Girardin SE, Boneca IG, Viala J, Chamaillard M, Labigne A, Thomas G, Philpott DJ, Sansonetti PJ 2003 Nod2 is a general sensor of peptidoglycan through muramyl dipeptide (MDP) detection. J Biol Chem 278:8869–8872. doi:10.1074/jbc.C200651200. 12527755

[41] Moreira LO, El Kasmi KC, Smith AM, Finkelstein D, Fillon S, Kim YG, Núñez G, Tuomanen E, Murray PJ 2008 The TLR2-MyD88-NOD2-RIPK2 signalling axis regulates a balanced pro-inflammatory and IL-10-mediated anti-inflammatory cytokine response to Gram-positive cell walls. Cell Microbiol 10:2067–2077. doi:10.1111/j.1462-5822.2008.01189.x. 18549453 PMC4966886

[42] Nilsen EM, Johansen FE, Jahnsen FL, Lundin KE, Scholz T, Brandtzaeg P, Haraldsen G 1998 Cytokine profiles of cultured microvascular endothelial cells from the human intestine. Gut 42:635–642. doi:10.1136/gut.42.5.635. 9659156 PMC1727090

[43] Nakamura N, Lill JR, Phung Q, Jiang Z, Bakalarski C, de Mazière A, Klumperman J, Schlatter M, Delamarre L, Mellman I 2014 Endosomes are specialized platforms for bacterial sensing and NOD2 signalling. Nature 509:240–244. doi:10.1038/nature13133. 24695226

[44] Stack J, Doyle SL, Connolly DJ, Reinert LS, O’Keeffe KM, McLoughlin RM, Paludan SR, Bowie AG 2014 TRAM is required for TLR2 endosomal signaling to type I IFN induction. J Immunol 193:6090–6102. doi:10.4049/jimmunol.1401605. 25385819 PMC4258402

[45] Brandt KJ, Fickentscher C, Kruithof EK, de Moerloose P 2013 TLR2 ligands induce NF-κB activation from endosomal compartments of human monocytes. PLoS One 8:e80743. doi:10.1371/journal.pone.0080743. 24349012 PMC3861177

[46] Underhill DM, Ozinsky A, Hajjar AM, Stevens A, Wilson CB, Bassetti M, Aderem A 1999 The Toll-like receptor 2 is recruited to macrophage phagosomes and discriminates between pathogens. Nature 401:811–815. doi:10.1038/44605. 10548109

[47] Schleifer KH, Kandler O 1972 Peptidoglycan types of bacterial cell walls and their taxonomic implications. Bacteriol Rev 36:407–477. 4568761 10.1128/br.36.4.407-477.1972 PMC408328

[48] Severin A, Tomasz A 1996 Naturally occurring peptidoglycan variants of *Streptococcus pneumoniae*. J Bacteriol 178:168–174. doi:10.1128/jb.178.1.168-174.1996. 8550412 PMC177635

[49] Dziarski R, Gupta D 2005 *Staphylococcus aureus* peptidoglycan is a Toll-like receptor 2 activator: a reevaluation. Infect Immun 73:5212–5216. doi:10.1128/IAI.73.8.5212-5216.2005. 16041042 PMC1201261

[50] Li Y, Efferson CL, Ramesh R, Peoples GE, Hwu P, Ioannides CG 2011 A peptidoglycan monomer with the glutamine to serine change and basic peptides bind in silico to TLR-2 (403-455). Cancer Immunol Immunother 60:515–524. doi:10.1007/s00262-010-0959-1. 21188584 PMC11028711

[51] Radin JN, Orihuela CJ, Murti G, Guglielmo C, Murray PJ, Tuomanen EI 2005 β-Arrestin 1 participates in platelet-activating factor receptor-mediated endocytosis of *Streptococcus pneumoniae*. Infect Immun 73:7827–7835. doi:10.1128/IAI.73.12.7827-7835.2005. 16299272 PMC1307033

[52] Fehrenbach ML, Cao G, Williams JT, Finklestein JM, Delisser HM 2009 Isolation of murine lung endothelial cells. Am J Physiol Lung Cell Mol Physiol 296:L1096–L1103. doi:10.1152/ajplung.90613.2008. 19304908 PMC2692810

[53] National Research Council 2011 Guide for the care and use of laboratory animals, 8th ed. National Academies Press, Washington, DC.

[54] Swiatlo E, Champlin FR, Holman SC, Wilson WW, Watt JM 2002 Contribution of choline-binding proteins to cell surface properties of *Streptococcus pneumoniae*. Infect Immun 70:412–415. doi:10.1128/IAI.70.1.412-415.2002. 11748210 PMC127640

[55] Bui NK, Eberhardt A, Vollmer D, Kern T, Bougault C, Tomasz A, Simorre JP, Vollmer W 2012 Isolation and analysis of cell wall components from *Streptococcus pneumoniae*. Anal Biochem 421:657–666. doi:10.1016/j.ab.2011.11.026. 22192687

[56] Kirchhausen T, Macia E, Pelish HE 2008 Use of dynasore, the small molecule inhibitor of dynamin, in the regulation of endocytosis. Methods Enzymol 438:77–93. doi:10.1016/S0076-6879(07)38006-3. 18413242 PMC2796620

