# Supplementary material for: Dissecting Bacterial Cell Wall Entry and Signaling in Eukaryotic Cells: an Actin-Dependent Pathway Parallels Platelet-Activating Factor Receptor-Mediated Endocytosis
Source: mBio. 2017 Jan 3;8(1):e02030-16. doi: 10.1128/mBio.02030-16 (PMC5210498; doi:10.1128/mBio.02030-16)
Supplement: Table S1 [file mbo006163129st1.docx]

**Antibodies**

| Target | Supplier | Antibody/ Product |
| --- | --- | --- |
| Clathrin heavy chain | Cell Signaling Technology | 2410 |
| Caveolin | BD Transduction Laboratories (San Jose, CA) | 610059 |
| Mouse LAMP2 | Developmental Studies Hybridoma Bank (DSHB, Iowa, IA) | GL2A7 |
| CD102 (ICAM-2) | SouthernBiotech (Birmingham, AL) | 1925-01 |
| FITC | Molecular Probes | A-11095 |
| Endophilin A2 | Santa Cruz Biotechnology (Dallas, Texas) | SC-365704 |

**Inhibitors**

| Inhibitor | Supplier | Product |
| --- | --- | --- |
| CV-3988 | Enzo Life Sciences (Farmingdale, NY) | BML-L103-0005 |
| Dynasore | Abcam (Cambridge, MA) | ab120192 |
| Cytochalasin D | Sigma-Aldrich | C8273 |
| Rhosin | EMD Millipore (Billerica, MA) | 555460 |
| Pirl1 | ChemBridge (San Diego, CA) | 5137877 |
| EHT1864 | Tocris Bioscience, Biotechne (Minneapolis, MN) | 3872 |
| Wortmannin | InvivoGen (San Diego, CA) | tlrl-wtm |
| EIPA | Sigma-Aldrich | A3085 |
| Bafilomycin A2 | InvivoGen (San Diego, CA) | Tlrl-baf1 |

**siRNAs**

| siRNA | Description | siRNA ID |
| --- | --- | --- |
| Silencer Negative Control No. 1 siRNA | Control siRNA | AM4611 |
| CLTC | Clathrin heavy chain | M-004001-00-0005 (Human)  M-063954-01-0005 (Mouse) |
| CAV1 | Caveolin 1 | M-003467-01-0005 (Human)  M-058415-01-0005 (Mouse) |
| Sh3gl1 | Endophilin A2 | M-048660-01-0005 (Mouse) |

All siRNAs used in this study were siGENOME SMARTpool siRNA (GE Dharmacon, Lafayette, CO), except the control siRNA was from Ambion .
